# Evaluation of AMG510 Therapy on *KRAS*-Mutant Non–Small Cell Lung Cancer and Colorectal Cancer Cell Using a 3D Invasive Tumor Spheroid System under Normoxia and Hypoxia

**DOI:** 10.3390/bioengineering9120792

**Published:** 2022-12-12

**Authors:** Meng Huang, Wei Hou, Jing Zhang, Menglan Li, Zilin Zhang, Xiaoran Li, Zaozao Chen, Cailian Wang, Lihua Yang

**Affiliations:** 1 Medical Center for Digestive Disease, The Second Affiliated Hospital of Nanjing Medical University, Nanjing 210009, China; 2 State Key Laboratory of Bioelectronics, School of Biological Science and Medical Engineering, Southeast University, Nanjing 210096, China; 3 Department of Oncology, School of Medicine, Southeast University, Nanjing 210009, China; 4 Institute of Medical Devices (Suzhou), Southeast University, Suzhou 215163, China; 5 Jiangsu Avatarget Biotechnology Co., Ltd., Suzhou 215163, China

**Keywords:** spheroid, invasion, imaging, AMG510, *KRAS*, non–small cell lung cancer, colorectal cancer

## Abstract

A 3D tumor spheroid has been increasingly applied in pharmaceutical development for its simulation of the tumor structure and microenvironment. The embedded-culture of a tumor spheroid within a hydrogel microenvironment could help to improve the mimicking of in vivo cell growth and the development of 3D models for tumor invasiveness evaluation, which could enhance its drug efficiency prediction together with cell viability detection. NCI-H23 spheroids and CT-26 spheroids, from a non–small cell lung cancer and colorectal cancer cell line, respectively, together with extracellular matrix were generated for evaluating their sensitivity to AMG510 (a *KRAS^G12C^* inhibitor) under normoxia and hypoxia conditions, which were created by an on-stage environmental chamber. Results demonstrated that NCI-H23, the *KRAS^G12C^* moderate expression cell line, only mildly responded to AMG510 treatment in normal 2D and 3D cultures and could be clearly evaluated by our system in hypoxia conditions, while the negative control CT-26 (*G12D*-mutant) spheroid exhibited no significant response to AMG510 treatment. In summary, our system, together with a controlled microenvironment and imaging methodology, provided an easily assessable and effective methodology for 3D in vitro drug efficiency testing and screenings.

## 1. Introduction

Two-dimensional (2D) cell culture—cells grow in a monolayer on petri dishes or culture flasks—has been widely used in drug development and therapeutics as one of the most employed pre-clinical in vitro methodologies during the past decades due to its simplicity, ease of handling, cost-effectiveness, good reproducibility, and its ability to grow a myriad of different cell types [1]. In the early 1990s, the National Cancer Institute (NCI) established the NCI60 cell panel to screen and examine compound effect on cell viability (60 human tumor cell lines derived from nine tumor types) [2]. However, in recent decades, plenty of publications have reported that the diffusion-limited distribution of oxygen, nutrients, metabolites, and signaling molecules cannot be mimicked in a 2D cell monolayer [3]. Thus, the current model makes it difficult to reproduce the true complexity and three-dimensional (3D) structure found in the human body. An investigation of genetically defined tumors indicated that oncogenic signals resembled gene expression profiles from spontaneous human cancers in 3D tissue instead of 2D culture [4]. Moreover, an analysis of the activity of compounds tested in pre-clinical in vivo and in vitro assays by the NCI60 cell line screen also revealed a very low success rate [5].

Spheroid-based cancer models help solid tumors grow in a 3D spatial conformation, and mimic the oxygen, nutrients, and stress of the tumor microenvironment. With development over time, these models could be classified into multicellular tumor spheroids (MCTS), tumor spheres, tissue-derived tumor spheres, and organ-specific multicellular spheroids [3]. Notably, the “organoid” is different from a “spheroid”, once also used as a momentous term in a spontaneously well-rounded 3D cancer structure, but now it is generally recognized as a special term referring to 3D structure models differentiated from stem cells or isolated organ progenitors [6]. MCTS are originally generated from single-cell suspension culture without a supply of an exogenous extracellular matrix (ECM) in the early stage, and they can be performed by various methods such as ultra-low attachment plates, hanging drop, magnetic levitation, 3D printing, roller tube, spinner flask, gyratory shaker, rotating-wall vessel and plate after coating (e.g., soft agar liquid overlay), matrix encapsulation, and matrix on top or embedded [7,8]. Among these methods, spheroid formation by seeding cells in ultra–low attachment plates has been applied more and more because of its simplicity—without any extra coating—and because it allows for microscopic visualization together with viability measurement in extended drug exposures [2,3].

A list of human carcinoma cell lines, based partly on the NCI-60-cell line screen, have been constructed into spheroids to set standardized large-scale drug test routines [9]. With developments in microscopy techniques, automatic mechanical engineering, and artificial intelligence, we were able to achieve dynamic, automated, quantitative imaging and analyses that are compatible with routine high-throughput pre-clinical studies. Previous research has reported that 40 tumor cell lines have been classified, and a highly malignant cell was chosen to exemplify the therapeutic effects of three specific molecularly targeted agents through either tumor spheroid growth or invasion [10].

Providing the tumor with an extracellular matrix (ECM), one of the major components for constructing the tumor microenvironment (TME), is important for 3D tumor modeling. Tumor spheroids with an ECM can provide direct measurement of cell invasiveness and may monitor to tumor evolution. Previous research in in vitro 3D models has indicated that ECM heterogeneity is crucial for controlling collective cell invasive behaviors and determining metastasis efficiency [11,12]. Scaffold-based spheroids not only support cell growth, as their tissue architecture and the ECM significantly influence tumor cell responses to TME signals [10], but they also provide an approach for cancer invasion research in an actual 3D environment rather than through 2D wound healing or the semi-3D transwell. Cell migration is a highly integrated multistep process that drives disease progression in cancer, and there is a lot of interest in investigating the process mechanisms so as to improve cancer therapy [13,14].

In this study, we constructed a 3D tumor-spheroid-ECM (TSE) model by creating a tumor spheroid embedded within an ECM to monitor the drug responses of two invasive cell lines to AMG510, an inhibitor that targets the G12C-mutant *KRAS* expression and that has achieved responses in some patients with non–small cell lung cancer (NSCLC) or colorectal cancer (CRC). Previous sensitivity testing of NCI-60 NSCLC cell lines found that the efficiency of AMG510 was associated with the *KRAS* expression and activation of the tested cells [15]. Similarly, in CRC cell lines, the AMG510 sensitivity was also affected by *KRAS* relative signaling expression [16]. In our research, both NCI-H23 and CT-26 cells were tested. NCI-H23 has been reported to have an intermediate sensitivity that is not sensitive enough to obtain an IC_50_ via cell viability detection while CT-26 is the most commonly used cell line in drug development. However, CT-26 is a *KRAS^G12D^*–mutant mouse CRC cell [17], so this spheroid to AMG510 treatment was set as a comparison. In consideration of the hypoxia promoting tumor progression, we also tested AMG510 sensitivity under both normoxia and hypoxia conditions [18,19,20]. Our cell viability, together with invasion analysis, contributed more details toward understanding the effect of AMG510 on 3D tumor models and proved that this 3D evaluation system could not only be an alternative for a standard 2D in vitro drug screening assay but could also provide higher fidelity and sensitivity than ordinary assays.

## 2. Materials and Methods

### 2.1. Cell Culture

NCI–H23 and CT-26 cells were cultured in RPMI 1640, and HT-29 was cultured in McCoy’s 5A medium supplemented with 10% heat-inactivated FBS and 1% P/S individually. All cells were maintained in standard culture conditions (37 °C in humidified air with 5% CO_2_) during cell proliferation. Cells were purchased from the cell bank, Shanghai Institute of Biochemistry and Cell Biology. All of the mediums, supplements, and other agents were purchased from ThermoFisher Scientific (Waltham, MA, USA).

In 2D testing, cells were deposited in a 96-well plate with 1 × 10^4^ cells per well for 2 days of culturing before drug treatment. The incubator was maintained at 21% O_2_ in normal testing conditions, but was adjusted to 1.5% O_2_ in hypoxic testing conditions.

### 2.2. Generation of Multicellular Spheroids

Cells expanded in cell culture flasks were detached with 0.25% trypsin/EDTA (Invitrogen) and were re suspended with complete cell culture medium at a final concentration of 10^5^ cells/mL. A quantity of 100 μL of this cell suspension (~10^4^ cells) was deposited in a 48-well plate (well diameter ~6 mm) with a U-shaped well bottom. This plate was provided by Avatarget kit with anti-adhesion treatment and a surrounding water channel for evaporation prevention (Avatarget). The plate was centrifuged at 500 rpm for 1 min and incubated at standard culture conditions for cell growth to obtain appropriate spheroids. After 4 days of culturing, the cell spheroids were pipetted and removed from the medium, and were then embedded in Matrigel (25 μL of 3.5 mg/mL Matrigel, 50 μL of Complete Culture Medium). After the cell spheroids had been embedded, the plate was incubated for 20 min in the incubator to solidify the gels. Thereafter, 150 μL of culture medium was overlaid on the hydrogel matrix in each well. The complete system was incubated for another 7–10 days both in normal testing conditions and hypoxic testing conditions. At least 6 spheroids were generated for each group.

### 2.3. Drug Treatment

Anti-tumor drug AMG510 was applied at the final concentration of 0.001 μM, 0.005 μM, 0.01 μM, 0.05 μM, 0.1 μM, 0.5 μM, 1 μM and 5 μM. The AMG510 (MedChemExpress, Shanghai, China) was dissolved according to the manufacturer’s instructions and 100× working solutions were prepared with DMSO. A 1‰ DMSO treatment was used as control.

### 2.4. Imaging

Digital images of the spheroids were acquired using an Avatarget SMART high content microscope with a 10× objective (Avatarget, Suzhou, China). The SMART system is a high content microscope with an artificial intelligence (AI)-based algorithm which could focus and centralize these spheroids automatically [21]. Four images were acquired for each spheroid and an integrated image was formed and processed with Avatarget SMART software. Generally, it could provide the diameter, roughness, and excess perimeter index (EPI) of the spheroids. Briefly, the EPI is defined as the ratio between the actual spheroid perimeter and the equivalent perimeter (Equation (1)).

(1) $$\mathrm{EPI}=\;\frac{P_{o\;}-P_{e}}{P_{e}}$$

(2) $$P_{e}=2\pi \sqrt{\frac{S}{\pi }}$$

where *P_o_*, *P_e_*, and *S* are the perimeter, equivalent perimeter, and area of the spheroid at the focal plane, respectively. The spheroid perimeter was located and drawn by the SMART software with optional deep learning algorithms, followed by the area of the spheroid at focal plane (S) which was measured with ImageJ. Then, the equivalent perimeter of the spheroid was calculated by Equation (2).

Meanwhile, two-dimensional cell observation and image acquirement were performed on an Olympus IX83 motorized microscope with a 10×/0.30 objective and a DFC450C camera (Olympus, Tokyo, Japan).

### 2.5. Spheroid Viability Testing

Spheroid viability was measured using an Alamar Blue assay. Cell viability was measured at days 1, 4, 7, and 10 after embedding the spheroids in Matrigel, respectively, or as indicated. Absorbance at 570 nm and 600 nm was measured using a Multiskan FC microplate photometer (ThermoFisher GO, Waltham, MA, USA). In a 2D assay, the cell viability was detected after 2 days of drug treatment.

### 2.6. Tumor Growth Inhibition Calculation

Tumor growth inhibition (TGI) was obtained referencing to an in vivo TGI [22], where the tumor weights were replaced into relative spheroid volumes (RTV) in Equation (3). Meanwhile, in order to eliminate the difference among spheroids, RTV was defined as the terminal volume relative to original volume (Equation (4)). In addition, the spheroid volume was calculated from the spheroid diameter outputted by the Avatarget software.

TGI = (RTV_control_ − RTV_treatment_)/RTV_control_ × 100% (3)

RTV = V_terminal_/V_original_ (4)

(5) $$V=\;\frac{4}{3}\pi {\left(\frac{d}{2}\right)}^{3}$$

RTV_control_ was the relative spheroid volume of the untreated group, RTV_control_ was the relative spheroid volume of the drug treated group, V_terminal_ was the volume of the spheroid at the last culture day (day 7 in this work) while V_original_ was the volume of the spheroid at the first culture day, and d was the spheroid diameter.

### 2.7. Statistical Analysis

Statistical analysis was performed using one-way ANOVA, two-way ANOVA or Student’s *t*-test with 95% confidence interval using the software GraphPad Prism. *p* < 0.05 was considered significantly different. Data were shown as means ± SEM. *: *p* < 0.05, **: *p* < 0.01, ***: *p* < 0.001, ns: not significant.

## 3. Results

### 3.1. Tumor Spheroid Construction

**Figure 1:**
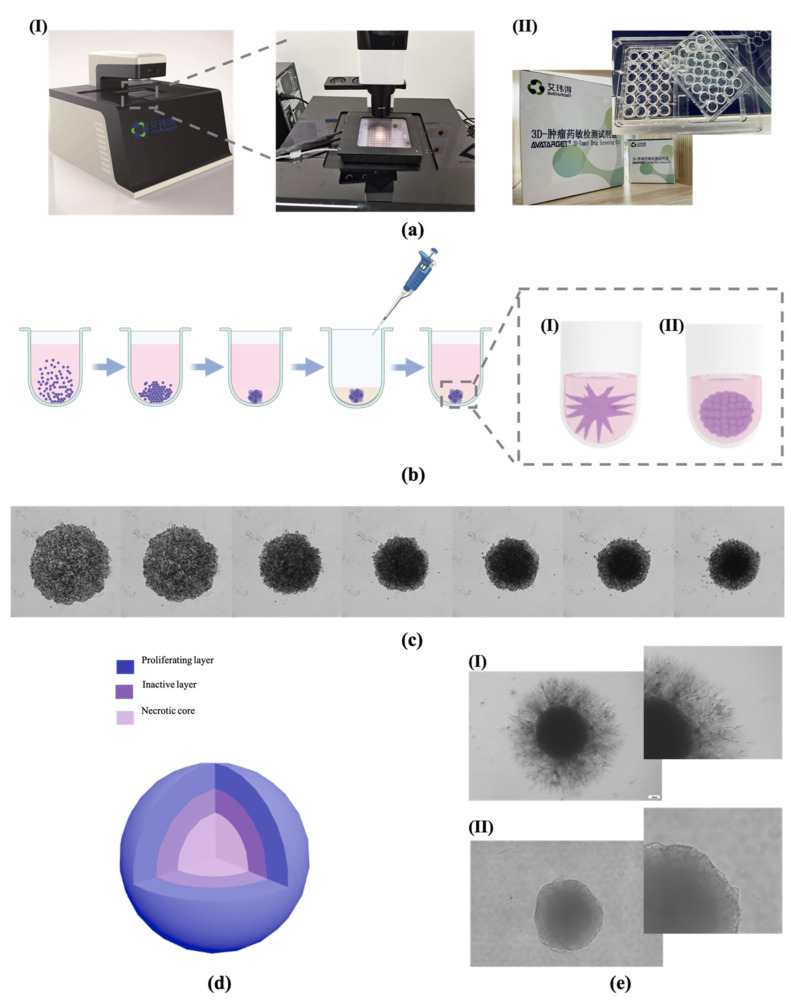
The testing principle and illustration of a tumor spheroid. (**a**) The equipment and kit used in tumor spheroid formation, and the optical observation and images capture. (**I**) High content microscope with artificial intelligence-based software and microscope-associated microenvironment controller. (**II**) Tumor spheroid formation kit with 48-well non-adhesive U-bottom plate. (**b**) The illustration of tumor spheroid culture timeline. (**I**) Invasive spheroid. (**II**) Non-invasive spheroid. (**c**) The bright field images of NCI-H23 spheroid cultured in the microenvironment controller within 48 h with photos taken every 8 h. (**d**) The different layers of a spheroid. (**e**) The morphological characteristics of invasive and less invasive tumor spheroids in a 7-day culture. (**I**) Spheroid of CT-26. (**II**) Spheroid of HT-29.

The formation of tumor spheroids was divided into two steps—the formation of spheroids and hydrogel embedding. All of the images taken during spheroid culture and drug treatment were performed through a high content imaging system with a microscope-associated microenvironment controller (Figure 1(aI)) and matching kit (Figure 1(aII)). As the schematic diagram shows (Figure 1b), first of all, the cancer cells were digested into a cell suspension and plated onto tailor-made 48-well non-adhesive U-bottom cell culture plates (provided by the kit; the picture is shown in Figure 1(aII)). The cells were precipitated to the bottom of the well by centrifugation, and then the tumor spheroids were cultured for four days. Next, we removed the cell culture mediums and added Matrigel to form the 3D matrix of tumor spheroids. Subsequently, some tumor spheroids showed an invasion ability (Figure 1(bI)), but others did not (Figure 1(bII)).

We used a high content imaging system that could apply automatic, intelligent, and real-time detection in order to record the process of tumor spheroid construction and invasion; the automated imaging system contained a microenvironment controller that could maintain the cell growth of tumor spheroids during the process of observation and imaging. In a 48 h observation (Figure 1c), the cell spheroids were aggregated and formed tighter spheroids in the first 24 h, and in the next 24 h, the cells in the spheroids gradually migrated outside to form a typical tumor structure—stratification of a proliferating layer, an inactive layer, and a necrotic core from the outside to the inside of the spheroid (Figure 1d).

Meanwhile, Figure 1(eI) had clearly shown that the CT-26 cancer cells migrated into the 3D Matrigel matrix, which means this was a significantly invasive cell line as the invasive spheroids always inhibit rough and unsharp boundaries [21]. Meanwhile, the HT-29 (a human colorectal cancer cell line) spheroid was a smooth spheroid and had no significant morphological changes in its construction process in the 3D matrix (Figure 1(eII)), which means there was low invasiveness. The invasion characteristics of the 3D reconstructed tumor spheroids in vitro were similar to those in vivo [23].

### 3.2. AMG510 Treatment to 2D Cultured NCI-H23 Cells

**Figure 2:**
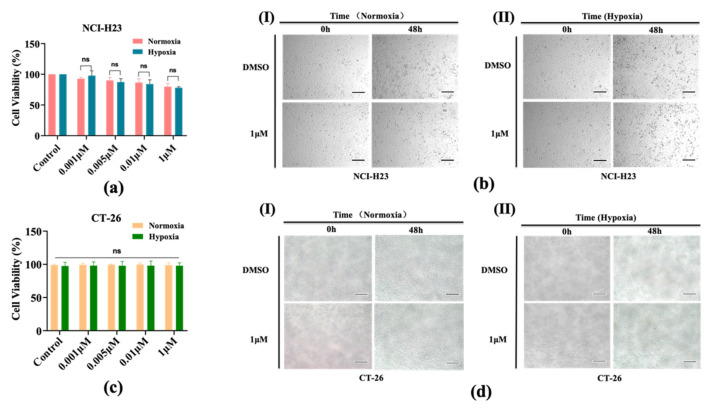
The AMG510 sensitivity of 2D NCl-H23 and CT-26 cells under normoxia and hypoxia conditions. (**a**) Cell viability of the NCl-H23 cell line treated with AMG510 for 48 h in 2D; the drug doses include 0.001 μM, 0.005 μM, 0.01 μM, and 1 μM. (**b**) The bright field images of NCl-H23 treated with AMG510 in 2D. (**I**) Normoxia. (**II**) Hypoxia. (**c**) Cell viability of the CT-26 cell line treated with AMG510 for 48 h in 2D; the drug doses include 0.001 μM, 0.005 μM, 0.01 μM, and 1 μM. (**d**) The bright field images of CT-26 treated with AMG510 in 2D; scale bar: 200 μM. (**I**) Normoxia. (**II**) Hypoxia. ns: not significant.

After 2 days of 2D cell culturing under different concentrations of oxygen (normoxia and hypoxia conditions), the NCl-H23 and CT-26 cells were treated under AMG510 therapy. In Figure 2a, although both groups were co-cultured with different concentrations of AMG510, neither of them showed a significant decrease in cell viability with increased drug doses, which indicated that the 2D culture of NCI-H23 had a low sensitivity to the AMG510 therapy. Our results showed that even at the highest concentration, the AMG510 could not inhibit cell viability to less than 50%. This result is consistent with a previous work [15], in which the IC_50_ of AMG510 could not be obtained on cells with moderated *KRAS G12C* mutant or without this mutant, and in that work, the highest concentration was 5 μM. For further convincing evidence, we can put more effort on image analysis as a supplement. Figure 2b shows how cells shared a completely different morphology with in vivo cells while the 3D culture of spheroids could mimic the in vivo tumor morphology and the Matrigel could form a more in vivo–relevant drug testing microenvironment [24]. Meanwhile, AMG510 exhibited no cytotoxicity to the CT-26 cells both under normoxia and hypoxia (Figure 2c,d). More images of the 2D cells are provided in Supplement Figure S1.

### 3.3. AMG510 Treatment to 3D NCI-H23 Spheroids

**Figure 3:**
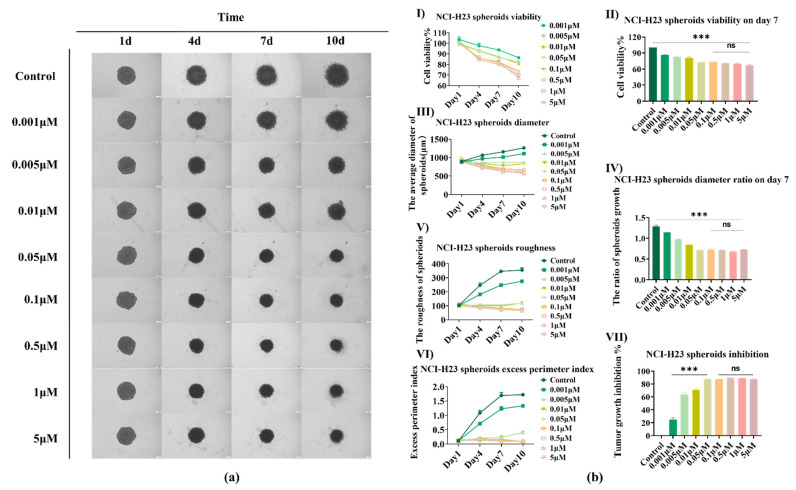
The AMG510 sensitivity of 3D NCl-H23 spheroids under normoxia conditions. (**a**) The bright field images of NCl-H23 spheroids treated with AMG510 under normoxia (21% O_2_); the drug doses include 0.001 μM, 0.005 μM, 0.01 μM, 0.05 μM, 0.1 μM, 1 μM, and 5 μM. (**b**) The cell viability and invasiveness analysis of NCl-H23 spheroids. (**I**) The cell viability of HCl-H23 spheroids treated with AMG510 within 10 days. (**II**) The cell viability of NCl-H23 spheroids treated with AMG510 on day 7; the data was normalized to control which was treated with 1% DMSO. (**III**) The diameters of NCl-H23 spheroids treated with AMG510 within 10 days. (**IV**) The diameters of HCl-H23 spheroids treated with AMG510 on the day 7 ratio to day 1. (**V**) The roughness of NCl-H23 spheroids treated with AMG510 within 10 days. (**VI**) The excess perimeter index (EPI) of NCl-H23 spheroids treated with AMG510 within 10 days. (**VII**) The tumor growth inhibition (TGI) of HCl-H23 spheroids treated with AMG510 on day 7. ***: *p* < 0.001, ns: not significant.

The sensitivity to AMG510 was enhanced under 3D spheroid conditions compared with 2D conditions, which is in keeping with a previous publication [15]. We first analyzed the images of a single spheroid under normoxia within 10 days, using the Avatarget SMART system. Significant volume reduction was found in samples with concentrations of AMG510 over 0.01 μM as shown in Figure 3a. In pre-clinical analysis, AMG510 treatment led to the regression of *KRAS* tumors [25,26]. The cell viability detected by the Alamar Blue assay (Figure 3(bI)) inhibited an obvious reduction with the increased concentration; the day 7 data comparison (Figure 3(bII)) enhanced the confidence level. Figure 3(bIII) showed the spheroid diameters within 10 days, and we can clearly find that if the drug concentration was over 0.01 μM, the spheroid was prevented from growing, and even had a decrease in size. Meanwhile, the 0.001 μM and 0.005 μM samples maintained their size over a few days, and the high dose sample (over AMG510 0.1 μM) had a near half spheroid diameter compared with the control at day 10. The day 7 data (Figure 3(bIV)) also presented a significant variation. As a result, although we cannot get the IC_50_ test data, we still succeeded in testing the AMG510 sensitivity. The Avatarget SMART system also analyzed the roughness (Figure 3(bV)) and the EPI (Figure 3(bVI)) data of the samples. As the former data showed a relatively stable result at day 7, we statistically analyzed the TGI of day 7 data and proved the efficient drug dose range (Figure 3(bVII)).The data showed a larger gap among the efficiency of AMG510 therapy with different drug doses. The concentrations over 0.01 μM indicated a more efficient therapeutic effect as these doses turned the tumor development into a stable disease. Although we found that a drug dose over 0.01 μM was efficient, we did not discover a dramatic difference among the efficient dose range. As a result, in the following test, we cut down the dose range and testing time.

### 3.4. The Sensitivity of NCI-H23 Spheroids to AMG510 Increases under Hypoxia

**Figure 4:**
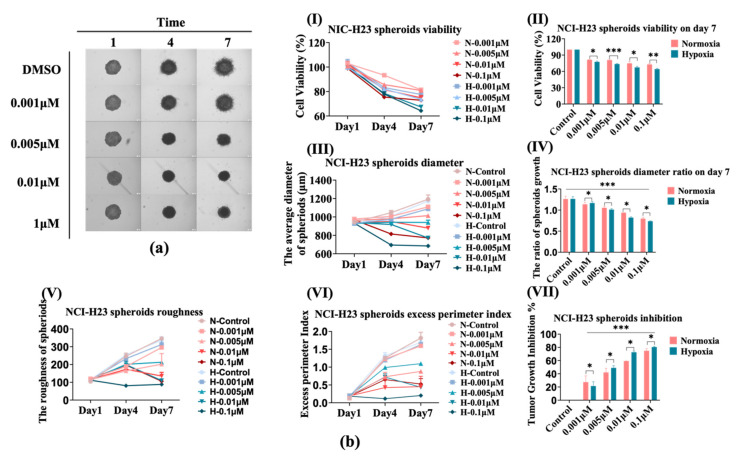
The AMG510 sensitivity of 3D NCI-H23 spheroids under hypoxia conditions compared with that of normoxia. (**a**) The bright field images of NCI-H23 spheroids treated with AMG510 under hypoxia (1.5% O_2_); the drug doses include 0.001 μM, 0.005 μM, 0.01 μM, and 0.1 μM. (**b**) The cell viability and size of NCI-H23 spheroids. (**I**) The cell viability of NCI-H23 spheroids treated with AMG510 within 7 days under normoxia and hypoxia conditions. (**II**) The cell viability of NCI-H23 spheroids treated with AMG510 on day 7; the data was normalized to control which was treated with 1% DMSO. (**III**) The diameters of NCI-H23 spheroids treated with AMG510 within 7 days under normoxia and hypoxia conditions. (**IV**) The diameters of NCI-H23 spheroids treated with AMG510 on the day 7 ratio to day 1. (**V**) The roughness of NCI-H23 spheroids treated with AMG510 within 7 days. (**VI**) The excess perimeter index (EPI) of NCI-H23 spheroids treated with AMG510 within 7 days. (**VII**) The tumor growth inhibition (TGI) of NCI-H23 spheroids treated with AMG510 on day 7. *: *p* < 0.05, **: *p* < 0.01, ***: *p* < 0.001.

Subsequently, we compared the effect of AMG510 treatment on 3D NCI-H23 spheroids under hypoxia compared with normoxia. As is shown in Figure 4a, the growth of spheroids was also inhibited by AMG510 that manifested in the decreased size and smoother edge along with the increased doses. The cell viability (Figure 4(bI)) presented stable reduction in 7-day culture, and the data analysis of day 7 evidenced this viability decline while it was significantly aggravated under hypoxia. A similar tendency was displayed in the diameter comparison (Figure 4(bIII)) which was exhibited distinctly on day 7 (Figure 4(bIV)); the diameters of these spheroids on the day 7 ratio to day 1 of themselves clearly indicated the contraction during AMG510 co-culturing and it was more visible than viability contrast. Similar to the previous 2D experiment, AMG510 still couldn’t obtain the IC_50_ through the cell viability assay even under hypoxia in 3D detection.

Notably, the NCI-H23 spheroids were more sensitive to AMG510 under hypoxia than under normoxia. To further evaluate the invasiveness of spheroids, we compared the roughness (Figure 4b,e) and the excess perimeter index (EPI, Figure 4(bVI) based on the spheroid boundary outputted by the Avatarget SMART software. NCI-H23 is an invasive cell whose spheroid roughness and EPI would increase with culture time (without treatment). However, AMG510 treatment attenuated this increase, which totally fit the size variation tendency similar to the TGI. Identically, the spheroid invasiveness was highly depressed under hypoxia conditions when the EPI was less than 0.5 in AMG510 0.1 μM treatment indicating a distinct invasion inhibition. In some cases, the cell line could be classified into a less invasive category [21]. The NCI-H23 TGI was calculated according to the spheroid diameter in Figure 4(bVII) seeing as it was a relative evaluation parameter that could decrease the original seeding difference among spheroids, and the trend was opposite to the diameter ratio (Figure 4(bIV)) which was consistent with the viability (Figure 4(bII)).

### 3.5. The Sensitivity of CT-26 Spheroids Does Not Change Significantly under Hypoxia

**Figure 5:**
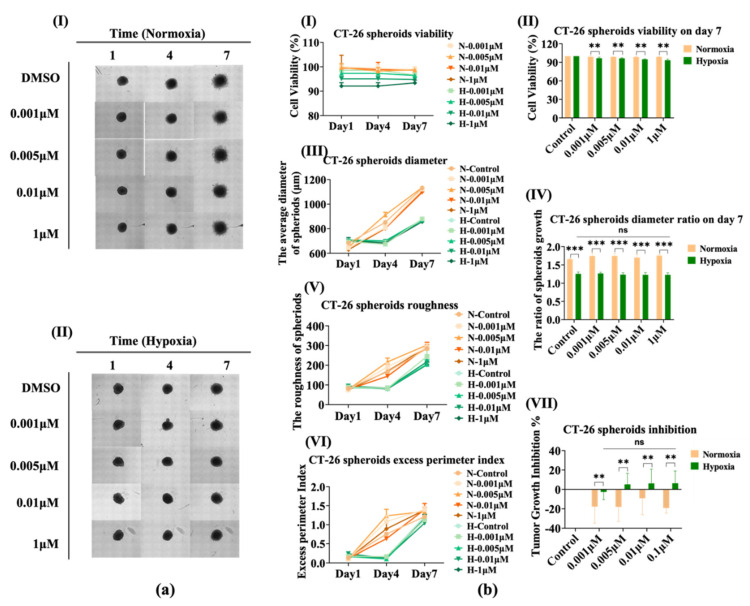
The AMG510 sensitivity of 3D CT-26 spheroids under hypoxia conditions compared with that of normoxia. (**a**) The bright field images of CT-26 spheroids treated with AMG510 under hypoxia (1.5% O_2_); the drug doses include 0.001 μM, 0.005 μM, 0.01 μM, and 0.1 μM. (**I**) The images obtained under normoxia. (**II**) The images obtained under hypoxia. (**b**) The cell viability and size of CT-26 spheroids. (**I**) The cell viability of CT-26 spheroids treated with AMG510 within 7 days under normoxia and hypoxia conditions. (**II**) The cell viability of CT-26 spheroids treated with AMG510 on day 7; the data was normalized to control which was treated with 1% DMSO. (**III**) The diameters of CT-26 spheroids treated with AMG510 within 7 days under normoxia and hypoxia conditions. (**IV**) The diameters of CT-26 spheroids treated with AMG510 on the day 7 ratio to day 1. (**V**) The roughness of CT-26 spheroids treated with AMG510 within 7 days. (**VI**) The excess perimeter index (EPI) of CT-26 spheroids treated with AMG510 within 7 days. (**VII**) The tumor growth inhibition (TGI) of CT-26 spheroids treated with AMG510 on day 7. **: *p* < 0.01, ***: *p* < 0.001, ns: not significant.

Similar to the testing of NCI-H23, we compared the effect of AMG510 treatment on 3D CT-26 spheroids under hypoxia compared with normoxia. As is shown in Figure 5a, the growth of spheroids was not inhibited under normoxia in which the cell migration was almost the same between control and the maximal dose up to 1 μM (Figure 5(aI)).However, the growth was visibly inhibited under hypoxia with no significant dependent dose (Figure 5(aII)). Even in the control treated with DMSO, the fierce migration of CT-26 cell was inhibited. The cell viability (Figure 5(bI)) presented no reduction in the 7-day culture both under normoxia and hypoxia, and the data analysis of day 7 (Figure 5(bII)) showed a stable difference between normoxia and hypoxia. Apparently, however, AMG510 still could not obtain the IC_50_ through the cell viability assay. There was no possible way to compare the cell growth using only the cell viability assay.

Then, based on the spheroid images, a similar tendency (but with a gapped difference) was displayed in the diameter comparison (Figure 5(b(III,IV))). The diameter contrast of these spheroids on the day 7 ratio to day 1 of themselves was more distinct between normoxia and hypoxia, and the difference was more significant than that in the NCI-H23 spheroids. As previously detected, CT-26 is a strongly invasive cell in the Matrigel culture system which is appropriate for observation of faint migration variation. Not surprisingly, the roughness (Figure 5(bV)) and the excess perimeter index (EPI, Figure 5(bVI)) maintained a rise under normoxia but decreased under hypoxia. Moreover, the CT-26 TGI in Figure 5(bVII) more distinctly reflected the effect of AMG510 and oxygen. The CT-26 cell was not inhibited and even grew within all of the AMG510 concentrations, whereas the cell growth was slightly inhibited by the AMG510 treatment with the deficiency of oxygen.

## 4. Discussion

AMG510 is well known as the first *KRAS^G12C^* inhibitor for clinical use [27]. In addition, AMG510 treatment can result in a pro-inflammatory tumor microenvironment (TME) and produce durable cures alone as well as in combination with immune checkpoint inhibitors [28]. AMG510 treatment results in inflammatory TME being highly sensitive to immunosuppression. The 2D culture of cells did not provide a TME which could be used in the test of AMG510 therapy. This also suggests that the use of the 3D culture system and the imaging analysis could provide great potential for complex drug screening or testing.

Cell invasion and metastasis are cancer hallmarks, and the cancer process is still an emerging field replete with major unanswered questions [29]. Cell viability alone cannot be enough in drug screening or therapy evaluation. For example, although microtubule-associated inhibitors (such as Paclitaxel and Docetaxel) strongly inhibited the growth in spheroid size and cell invasiveness, they did not significantly inhibit spheroid viability [21]; or moreover, the cell was not sensitive enough to obtain IC_50_ in detection of the AMG510 treatment applied to NCI-H23, et al. [15]. Additionally, cell migration is a critical event in tumor invasion and metastasis, and transwell and wound-healing assays are the most common methods used to study cell migration. However, the most current methods only detect cell migration at the 2D level in vitro, which cannot mimic the in vivo physiological environment and cannot detect the true situation of cell migration in vivo [30]. Recently, 3D cell migration in hydrogel-assembled microenvironments is becoming a trend, and the tumor spheroid culture in hydrogel would be a favorable way to evaluate tumor invasion as a supplement to viability detection.

However, the limited measurements and analyses of 3D spheroids have restricted their widespread application. Either spheroid boundary or invasion quantitative analysis is troublesome in invasion evaluation during the drug treatment. In high-throughput detection, a high content microscope with an AI-based algorithm might provide great help in the evaluation of tumor spheroid invasiveness both from cell lines and primary tumor cells [21]. In this research, we used a high content microscope (Avatarget) and software (SMART) to easily construct and analyze the 3D spheroids.

It is well known that mutations in *KRAS* play a critical role in metabolic reprogramming in multiple cancers, including lung cancer and colorectal cancer. Cancer cells rewire their metabolic programs in response to changes in the tumor microenvironment and in oncogenic signals such as an activating *KRAS* mutation [31] while AMG510 is a *KRAS* inhibitor targeting *G12C*-mutant *KRAS*. Our results verified the fact that AMG510 treatment obviously worked on the *G12C*-mutated NCI-H23 spheroids, but its effect was indistinguishable on the *G12D*-mutated CT-26 spheroids. According to results from a clinical trial called CodeBreak-100 including 126 participants, approximately 37% with previously treated advanced NSCLC (with *KRAS^G12C^* mutations) experienced substantial tumor size reductions. In addition, tumors were completely diminished in two trial participants [32]. The clinical trial results indicate that it is possible to use 3D spherical models, for which we were able to get similar results in vitro.

Hypoxia is a common feature of solid tumors and it is associated with cancer aggressiveness, treatment resistance, and poor prognosis. Mutant *KRAS* modulates the metabolic plasticity of cancer cells conferring a growth advantage during hypoxia [20]. In addition, multiple studies have demonstrated that under hypoxia, cancer cells engage metabolic adaptation strategies to survive and grow by activating a relevant gene expression program through *HIF-1α* [33,34] whereby the oxygen-sensitive hypoxia inducible factor (*HIF*) transcriptional regulators *HIF-1α* and *HIF-2α* are overexpressed in many human NSCLC [18]. We considered that the upgraded sensitivity of NCI-H23 spheroids to AMG510 under hypoxia may be because the *KRAS* modulation is more active under hypoxic conditions that result in an enhanced inhibition of AMG510.

**Figure 6:**
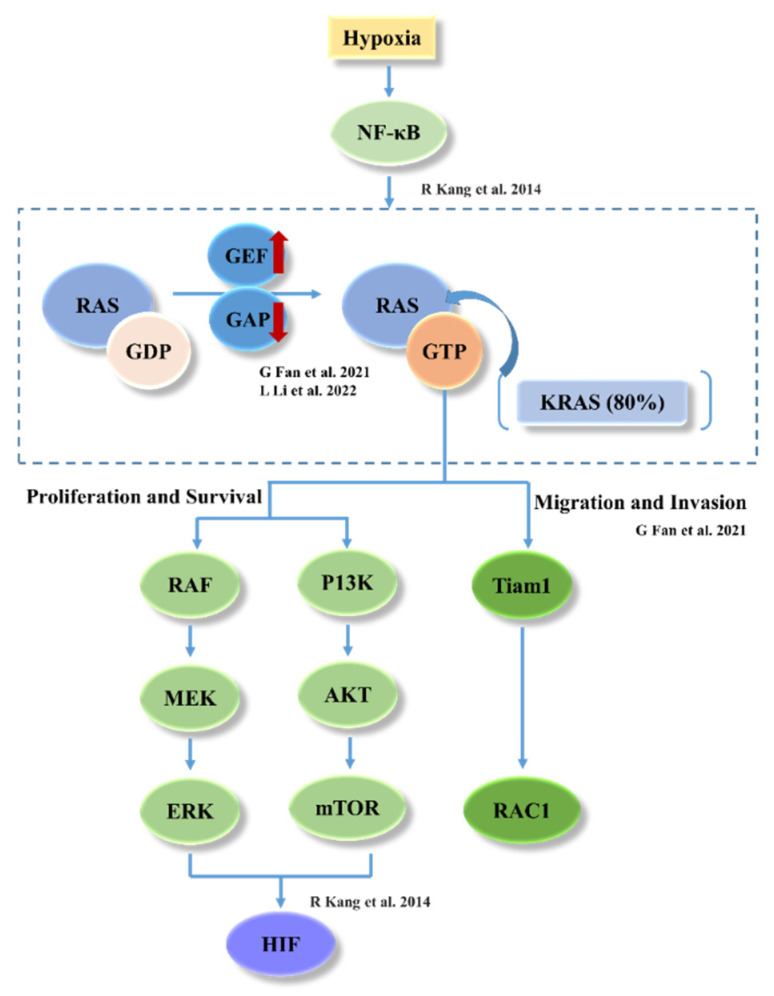
The signaling pathway of *KRAS-HIF* under hypoxia [19,36,37].

As expected, the CT-26 spheroids exhibited no sensitivity to AMG510 under normoxia and hypoxia seeing as CT-26 is a *KRAS^G12D^* mutant cell line, and previous research has indicated that *HIF-1α* was induced by *KRAS^G12V^* signaling at the transcription level in colorectal cancer [35]. Interestingly, the CT-26 cell growth was strongly inhibited under hypoxia but exhibited a particularly feeble sensitivity to AMG510, which was barely observed by other detection processes such as cell viability. This may be because *KRAS^G12D^* differs from *KRAS^G12C^* in only one amino acid residue (*12C* vs. *12D*), and it retains the GTP/GDP-binding small protein (GTPase) activity indicating that the active form of *KRAS^G12D^* may possess the site to which inhibitors bind [36], and that the hypoxia conditions magnified the interaction. The activity of *RAS* (*KRAS* mutations accounting for approximately 80% of all *RAS* mutations) can be controlled by a GTP/GDP cycle which is negatively regulated by a GTPase-activating protein, thus leading to the activation of downstream signaling pathways, for example, *RAS-RAF-MEK-ERK* or *PI3K-AKT*, the two main pathways that contribute to *HIF-1α* [37] (Figure 6). However, it is still unknown if the difference is consistent with the *K-RAS G12D* mutation in CT-26. Therefore, more types of cells should be studied in future work.

In addition to lung and colorectal cancer, oncogenic *KRAS* could also facilitate *HIF-1α* activation and promote pancreatic tumor growth under hypoxic conditions [19]. Extended to a clinical application, a comparison of the relevance with clinical observations showing that patients with CRC derive less benefit compared with those with NSCLC from a *KRAS* inhibitor [38], may indicate that a hypoxia-dependent complex signaling pathway should be taken into consideration in *KRAS*-mutant cancer research.

## 5. Conclusions

In recent decades, 3D tumor models have made significant improvements and have facilitated drug screening by providing more mimetic tumor structure and growth microenvironments. The combination with hydrogel broadened its invasiveness detection that could provide more details in drug evaluation. Our attempt in the detection of AMG510 to the moderated expression *KRAS*-mutant cell line NCI-H23 and CT-26 showed that this system could precisely evaluate the drug effect, and that it was suitable in distinguishing the slight difference between normoxia and hypoxia culture conditions. Moreover, the invasiveness evaluation based on 3D cell migration in hydrogel was analyzed by an artificial intelligence algorithm, which provided an extra dimension for tumor characterization and drug efficacy analysis. In summary, the methodology provided here could be an ideal in vitro model in drug screening or therapy evaluation for its convenience in operation and for its high efficiency.

## Data Availability

Not applicable.

## Supplementary Materials

The following supporting information can be downloaded at: https://www.mdpi.com/article/10.3390/bioengineering9120792/s1, Figure S1. The bright field images of NCl-H23 and CT-26 treated with AMG510 in 2D, the drug doses include 0.001 μM, 0.005 μM, 0.01 μM, 1 μM. (a) NCI-H23 normoxia. (b) NCI-H23 hypoxia. (c) CT-26 normoxia. (d) CT-26 hypoxia. Scale bar: 200 μM.

## Abbreviations

KRAS	Kirsten ratsarcoma viral oncogene homolog
ECM	Extracellular matrix
TME	Tumor microenvironment
TSE	3D tumor-spheroid-ECM
NSCLC	Non–small cell lung cancer
CRC	Colorectal cancer
EPI	Excess perimeter index
TGI	Tumor growth inhibition
RTV	Relative spheroids volumes
HIF	Hypoxia inducible factor
