# Supplementary material for: Evaluation of AMG510 Therapy on KRAS-Mutant Non–Small Cell Lung Cancer and Colorectal Cancer Cell Using a 3D Invasive Tumor Spheroid System under Normoxia and Hypoxia
Source: Bioengineering (Basel). 2022 Dec 12;9(12):792. doi: 10.3390/bioengineering9120792 (PMC9774149; doi:10.3390/bioengineering9120792)
Supplement: Supplementary file 1 [file bioengineering-09-00792-s001.zip › bioengineering-2075966-supplementary.pdf]

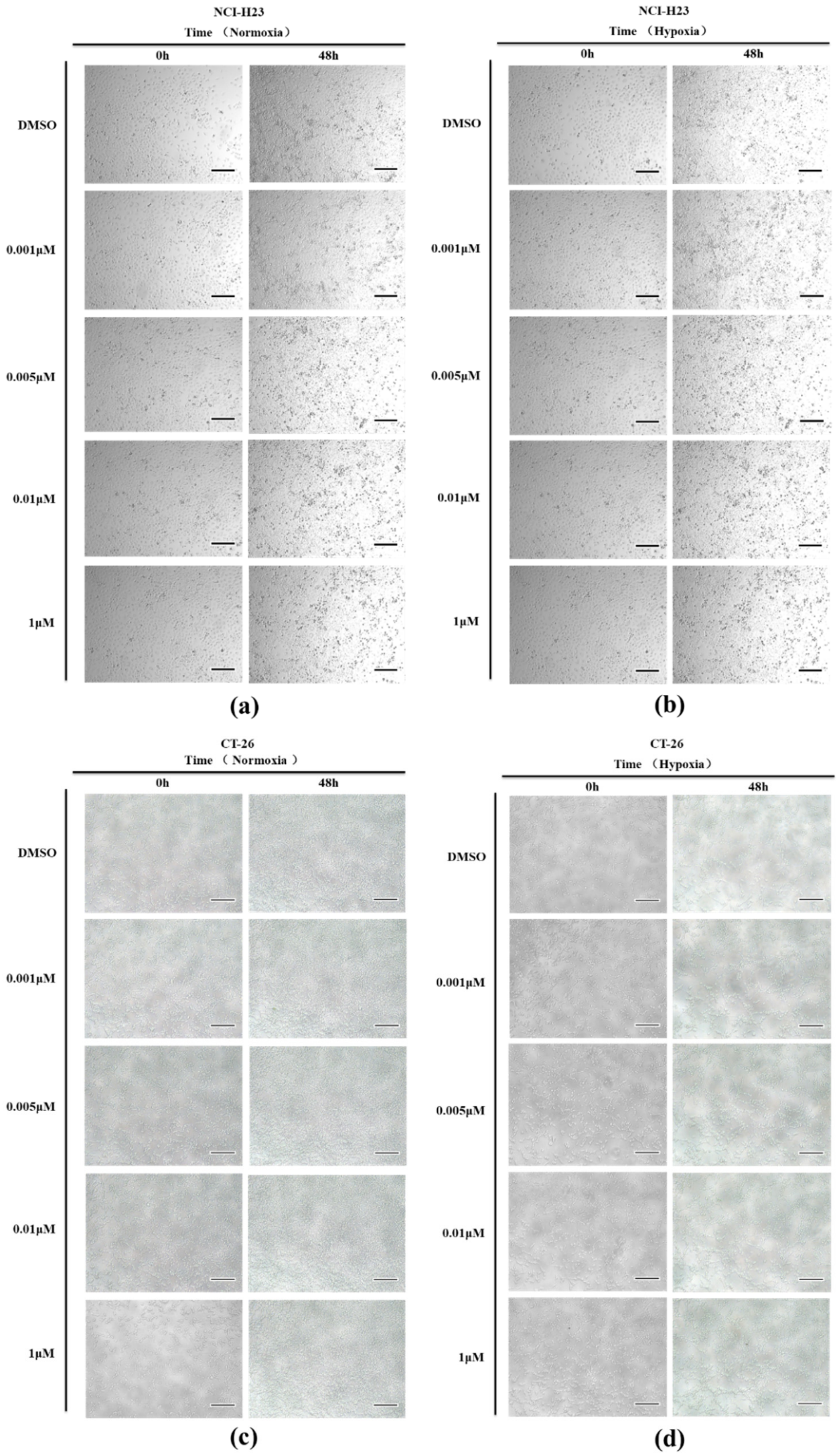

Figure S1. The bright field images of NCl-H23 and CT-26 treated with AMG510 in 2D, the drug doses include 0.001μM, 0.005μM, 0.01μM, 1μM. (**a**) NCI-H23 normoxia. (**b**) NCI-H23 hypoxia. (**c**) CT-26 normoxia. (**d**) CT-26 hypoxia. Scale bar: 200μM.
